# Fate of HIV-1 cDNA intermediates during reverse transcription is dictated by transcription initiation site of virus genomic RNA

**DOI:** 10.1038/srep17680

**Published:** 2015-12-03

**Authors:** Takao Masuda, Yoko Sato, Yu-Lun Huang, Satoshi Koi, Tatsuro Takahata, Atsuhiko Hasegawa, Gota Kawai, Mari Kannagi

**Affiliations:** 1 Department of Immunotherapeutics, Graduate School of Medical and Dental Sciences, Tokyo Medical and Dental University, Yushima, Bunkyo-ku, Tokyo 113-8519, Japan; 2 Department of Life and Environmental Sciences, Faculty of Engineering, Chiba Institute of Technology, 2-17-1 Tsudanuma, Narashino-shi, Chiba 275-0016, Japan

## Abstract

Retroviral reverse transcription is accomplished by sequential strand-transfers of partial cDNA intermediates copied from viral genomic RNA. Here, we revealed an unprecedented role of 5′-end guanosine (G) of HIV-1 genomic RNA for reverse transcription. Based on current consensus for HIV-1 transcription initiation site, HIV-1 transcripts possess a single G at 5′-ends (G1-form). However, we found that HIV-1 transcripts with additional Gs at 5′-ends (G2- and G3-forms) were abundantly expressed in infected cells by using alternative transcription initiation sites. The G2- and G3-forms were also detected in the virus particle, although the G1-form predominated. To address biological impact of the 5′-G number, we generated HIV clone DNA to express the G1-form exclusively by deleting the alternative initiation sites. Virus produced from the clone showed significantly higher strand-transfer of minus strong-stop cDNA (-sscDNA). The *in vitro* assay using synthetic HIV-1 RNAs revealed that the abortive forms of -sscDNA were abundantly generated from the G3-form RNA, but dramatically reduced from the G1-form. Moreover, the strand-transfer of -sscDNA from the G1-form was prominently stimulated by HIV-1 nucleocapsid. Taken together, our results demonstrated that the 5′-G number that corresponds to HIV-1 transcription initiation site was critical for successful strand-transfer of -sscDNA during reverse transcription.

Reverse transcription of single-stranded viral genomic RNA into double-stranded DNA is a characteristic feature of retroviruses including human immunodeficiency virus type 1 (HIV-1). The reverse transcription is catalyzed by retroviral enzyme, reverse transcriptase (RT). Mature form of HIV-1 RT is a heterodimer consisting of p66 and p51 subunits^1^. The p66 subunit contains DNA polymerase and RNase H domains and exerts both of catalytic activities^2^,^3^. The p51 subunits contains only polymerase domain. Recently, it has been reported that p51 subunit orients the RNA strand at the RNase H active site in the p66 subunit, indicating a critical roles of p51 for catalytic functions of RT^4^,^5^,^6^,^7^.

Soon after entry into cells, the minus-strand cDNA corresponding to the R-U5 region of viral RNA was firstly synthesized to generate minus-strand strong stop cDNA (-sscDNA). In case of HIV-1, tRNA^lys3^ was used to initiate -sscDNA synthesis as a primer^8^,^9^,^10^,^11^. Then RNase H within RT digests the R-U5 region of viral RNA duplexed with -sscDNA. Using complementarity of the R region, resultant -sscDNA is transferred to 3′-end of viral RNA (1^st^ strand-transfer). After the 1^st^ strand-transfer, extension of -sscDNA occurs to copy the rest of viral RNA, generating a new duplex of minus strand cDNA and viral RNA. Then, RT-associated RNase H digested RNA in the duplex. A purine-rich sequence (PPT) which is resistant to RNase H, serves as a next primer to synthesis of plus-strand strong stop cDNA (+sscDNA). Resultant +sscDNA forms a duplex with PBS region of the tRNA primer. After digestion of the PBS region by the RNase H, +sscDNA is transferred to the 3′ end of plus-strand DNA by using complementarity of the PBS sequence (2^nd^ strand-transfer). Finally, +sscDNA functions as a primer to synthesize plus-strand cDNA. Thus, at least two strand-transfers of partially synthesized cDNA intermediates are required to generate complete form of viral DNA (for review see refs 12, 13, 14).

The strand-transfer events have been analyzed extensively by using artificial substrates that mimic cDNA intermediates of the reverse transcription^12^,^13^. It has been noticed that substantial amount of aberrant cDNA species were generated during the strand-transfer events^15^. The presence of the large stem-loop structure in the R region (called TAR) inhibits strand-transfer and is correlated with extensive synthesis of heterogeneous DNAs formed by self-priming of -sscDNA^16^. HIV-1 nucleocapsid protein (NC) drastically reduced self-priming and dramatically increases the efficiency of strand-transfer by destabilizing the secondary structures of TAR. NC exerts these functions through its nucleic acid chaperone activity to promote helix destabilization and/or hybridization (for review see ref. 17).

Generation of abortive cDNA intermediates during revere transcription is lethal not only to virus and but also to host cells. Recent studies demonstrated that the cDNA intermediates of HIV-1 during reverse transcription induced massive cell death^18^ in CD4^+^ T cells through their recognition by cellular sensor^19^. These studies have evoked a novel linkage between the abortive cDNA generation and HIV-1 pathogenesis. However, intrinsic mechanism for the abortive cDNA generation and its regulation are largely unknown.

On the other hand, retrovirus genomic RNAs in virus particles originate from viral transcripts, which were expressed from provirus DNA by cellular RNA polymerase II. Nucleotide at 5′-end of retrovirus transcripts corresponds to the transcription initiation site, which is located within the U3/R junction of proviral DNA. At the U3/R junction of HIV-1 proviral DNA, there is a conserved tract consisting of three guanosines (GGG-tract). Current consensus for the initiation site is at the last G in the GGG tract (http://www.hiv.lanl.gov, HIV Sequence Compendium 2014), generating HIV-1 transcripts with one G at their 5′-ends^20^. It also has been reported that HIV-1 transcripts initiated at other Gs within the GGG tract^21^,^22^. Since 5′-ends of viral genomic RNA define 3′-ends of -sscDNA, we reasoned that the 5′-end nucleotides of viral RNA would be critical for successful strand-transfer of -sscDNA (1^st^ strand-transfer) and subsequent elongation step.

In this study, we found that HIV-1 RNAs with different numbers of G at their 5′-ends (5′-G) were expressed within cells, which were persistently infected with HIV-1 and transfected with HIV-1 molecular clone DNA. These findings prompted us to address possible role(s) of the G nucleotides at 5′-end of HIV-1 genomic RNA during reverse transcription events. Through quantitative and qualitative analysis of cDNA intermediates, which were generated within infected cells and *in vitro* reaction, we revealed that the 5′-G that corresponded to initiation site of HIV-1 genomic RNA might have a critical role for successful 1^st^ strand-transfer during reverse transcription.

## Results

### Analysis of 5′-end nucleotides of HIV-1 transcripts

**Figure 1:**
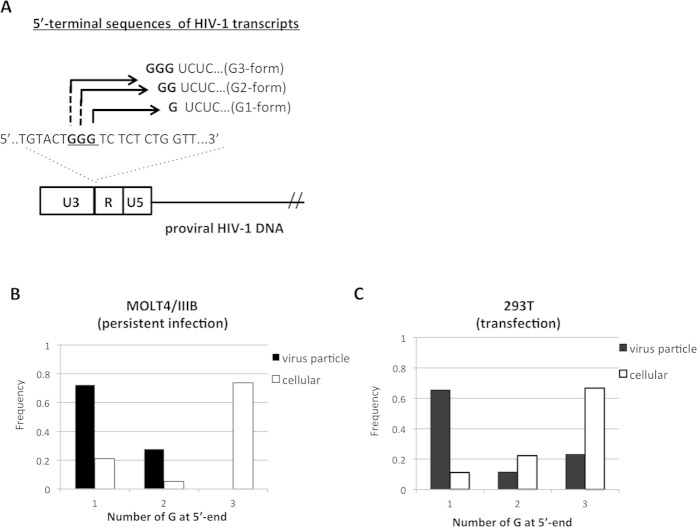
Analysis of 5′-end nucleotides of HIV-1 RNA. (**A**) HIV-1 transcription initiation sites were located within the GGG tract (underlined) at the U3/R junction of HIV-1 provirus DNA. Nucleotide sequences of HIV-1 transcripts initiated at each G within the GGG tract (G1-, G2- or G3-form) were shown. (**B**) mRNA fraction from MOLT4/IIIB cells (cellular) or from virus particles (virus particle) was subjected to the 5′-RACE analysis. Value was shown as frequency of each number of 5′-G among ~30 clones analyzed. (**C**) 293T cells were transfected with HIV-1 molecular clone (pNL-luc∆env) together with VSV-G expression vector. At 48 h post-transfection, mRNA was isolated from 293T cells or from virus particles and subjected to the 5′-RACE analysis. Value was shown as frequency of each number of 5′-G among ~30 clones analyzed.

The 5′-end nucleotide of HIV-1 RNA is defined by transcription initiation site, which is located at U3/R junction of integrated HIV-1 DNA. HIV-1 DNA encodes three nucleotides of guanosine (GGG tract) at the U3/R junction (Fig. 1A). The last G in the GGG tract is currently proposed as the consensus transcription initiation site for HIV-1 (http://www.hiv.lanl.gov, HIV Sequence Compendium 2014). Based on this consensus site, HIV-1 transcripts have one G at 5′-end (5′-G). However, alternative initiation sites within the GGG tract also have been reported previously^20^,^21^,^22^, suggesting expression of HIV-1 transcripts with additional G(s) at 5′-ends. To address frequency of HIV-1 transcripts that use each alternative initiation site, we determined 5′-end nucleotide sequences of HIV-1 transcripts expressed in human T cells persistently infected with HIV-1 (MOLT-4/IIIB)^23^. Firstly, we confirmed that proviral HIV-1 DNAs in MOLT-4/IIIB cells possessed intact sequences of the U3/R boundary including the GGG tract. Then, we isolated mRNA from MOLT-4/IIIB cells (cellular) and from virus particles released into the culture supernatant (virus particle). Each fraction of mRNA was treated with tobacco acid pyrophosphatase (TAP) to remove the 7-methylguanosine (cap) and subjected to the 5′-rapid amplification of cDNA ends (5′-RACE) analysis. The 5′-RACE analysis showed that HIV-1 transcripts with three Gs at 5′-ends (G3-form) were dominantly (~68%) expressed in infected cells. In contrast, HIV-1 transcripts with two Gs (G2-form) or one G (G1-form) was ~18% or ~13%, respectively (Fig. 1B: cellular). These results suggested that HIV-1 transcripts were expressed from alternative initiation sites, which was 1 or 2 nt upstream of the consensus site. In contrast to cellular mRNA fraction, HIV-1 genomic RNA purified from virus particles showed high frequency (~70%) of the G1-form (Fig. 1B, virus particle), suggesting preferential packaging of the G1-form of transcripts. Similar results were obtained by examining mRNA fraction isolated from 293T cells after transfection of HIV-1 molecular clone, pNL43luc∆env^24^. For preparation of virus particle, 293T cells were co-transfected with pNL43luc∆env and VSV-G-expression vector, pHCMVG^25^. Here again, we found that the G3-form predominated over the other forms of HIV-1 transcripts in the transfected cells (Fig. 1C:cellular). In contrast, the G1-form predominated in the virus particles (~62%). However, the G2- and G3-forms were also detected in virus particles at a low frequency (Fig. 1C: virus particle).

### Modification of HIV-1 clone DNA to produce the G1-form of transcripts dominantly

**Figure 2:**
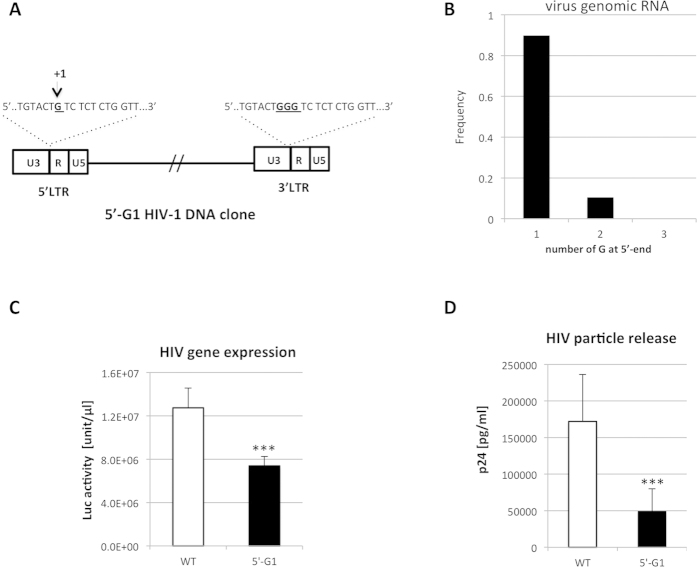
Generation of HIV-1 from DNA clone with a deletion mutation at the GGG tract. Construct of the modified pNL-luc∆env clone (5′-G1) was schematically depicted. In the 5′-G1 clone, two Gs were deleted in the GGG tract at 5′ LTR, while the GGG tract in the 3′ LTR was kept intact. Expected transcription initiation site was indicated as +1 (**A**). 293T cells were transfected with modified pNL-luc∆env vector (5′-G1) together with VSV-G expression vector (pMD.G). At 48 h post-transfection, mRNA was isolated from virus particles and subjected to the 5′-RACE analysis. Value was shown as frequency of each number of the 5′-G among ~30 clones analyzed (**B**). The levels of virus gene expression and virus particle release were determined by measuring luciferase activity (Luc activity) in the cell lysate (**C**) and HIV-1 p24 concentrations in the culture supernatant (**D**). Experiments were performed three times and representative experiments with means ± SE in duplicate assays are shown. Statistical significance was assessed by student *t* analysis (***p < 0.001).

We detected the G2- and G3-form of HIV-1 genomic RNA in virus particles, in addition to predominant G1-form. These findings prompted us to address a biological impact of the 5′-G during HIV-1 replication, especially at reverse transcription, since it defines the 3′-ends of -sscDNA. To address this point, we generated modified HIV-1 clone DNA to express the G1-form of transcripts exclusively (5′-G1 clone), by introducing a deletion of two Gs in the GGG tract at 5′ LTR of pNL43luc∆env (Fig. 2A). We kept the GGG tract in the 3′ LTR intact for the mutant clone. After transfection of 293T cells with the 5′-G1 clone together with VSV-G-expression vector, the 5′-G number of HIV-1 RNAs in mRNA fraction of cellular or virus particle was analyzed as described in Fig. 1. We confirmed that HIV-1 genomic RNAs in virus particle from the 5′-G1 clone were almost the G1-form (Fig. 2B). Curiously, the G2-form was detected in the virus particle with a low frequency. It has been reported that the provirus transfection with VSV-G distorts the result since it causes the amplification of virus by re-infection^26^,^27^. The GGG motif was generated during reverse transcription by copying the corresponding sequences at 3′-terminal region of virus RNA. This region was kept intact in the 5′-G1 clone (Fig. 2A). Therefore, the G2-form of viral RNA (Fig. 2B) might be generated through the re-infection of virus that contained the G1-form RNA. In contrast, truncated forms of the transcripts were often detected within cells after transfection of the 5′-G1 clone. Except these truncated forms, HIV-1 transcripts were exclusively the G1-form within the cells. We also noticed significant reductions in viral gene expression (Fig. 2C) and particle release (Fig. 2D) after transfection of the 5′-G1 clone. Truncated form of viral mRNA might relate to this reduction. The reduction of viral gene expression and particle release by the 5′-G1 mutations was also reproduced when the transfection experiment of pNL43luc∆env vector was performed without VSV-G vector, we can not exclude the possibility that the reduction might be due to the different transfection efficiency of each vector (data not shown). Although the precise mechanism for impact of the 5′-G1 mutation on HIV-1 gene expression remained to be determined, these results suggested a critical role of the GGG tract in proviral DNA for optimal viral gene expression. Nonetheless, we can generate virus, which packaged the G1-form of HIV-1 genomic RNA at higher frequency (>90%, Fig. 2B) compared with that (~65 %) in control virus (Fig. 1C).

### Evaluation of the 5′-G during early events of HIV-1 infection cycle

**Figure 3:**
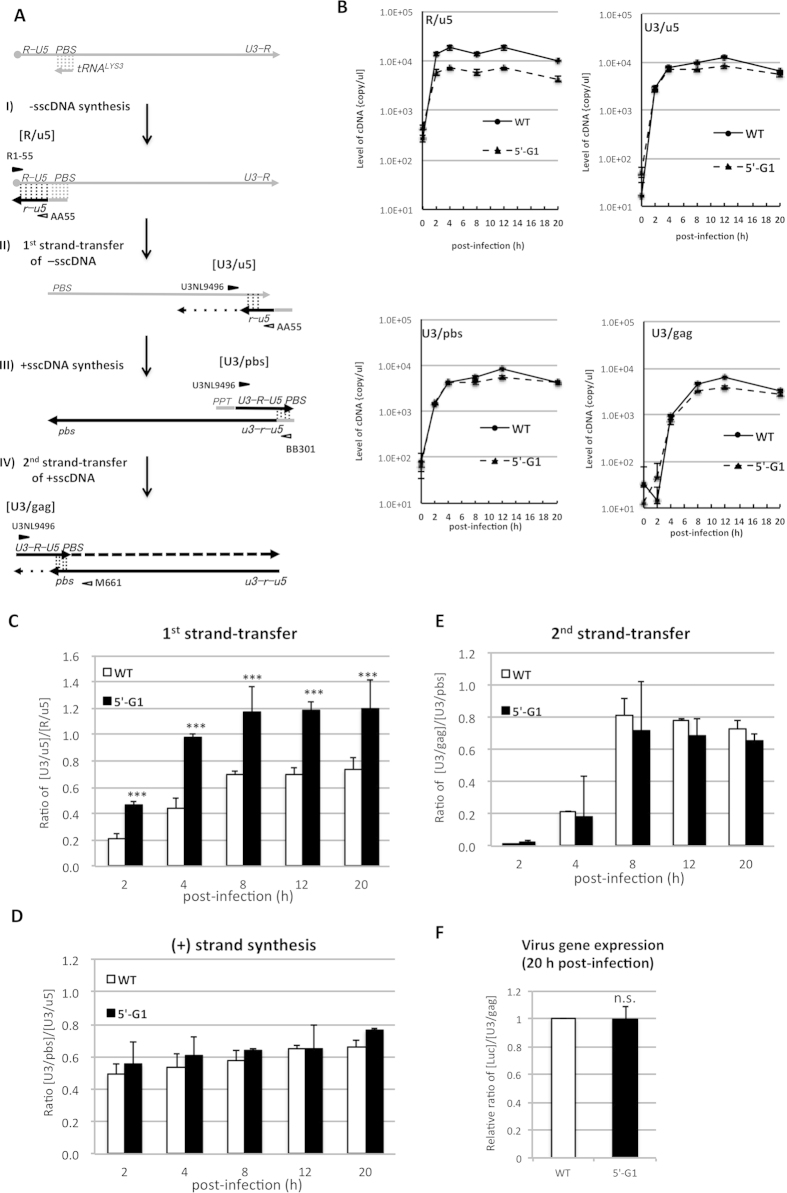
Evaluation of the 5′-G on strand-transfer events of reverse transcription within cells. Schematic flow of the reverse transcription was depicted. Gray or black lines represent HIV-1 genomic RNA or cDNA intermediates, respectively (**A**). Locations of the primers (R1-25, AA55, U3NL9496, BB301 and M661) to detect HIV-1 cDNA intermediates (R/u5, U3/u5, U3/pbs and U3/gag) were indicated. The minus-strand corresponding to the U3, R, U5 and PBS regions were shown by small characters of u3, r, u5 and pbs, respectively. For infection experiment, virus fraction containing ~80 ng of p24 was inoculated into 293T cells (1 × 10^6^ cells). Total DNA was extracted from the infected cells at 2, 4, 8, 12 or 20 h post-infection. An aliquot of each virus preparation was incubated at 65°C for 30 min and used as a heat-inactivated control. Each sample was subjected to the qPCR analysis with the primer pairs to measure the amount of cDNA intermediates with primer pairs specific for the R/u5 region of HIV-1 (M667/AA55), the U3/u5 region (U3NL9496/AA55), the U3/pbs region (U3NL9496/BB301) or the U3/gag region (U3NL9496/M661). Values were shown as copy numbers in 1μ of DNA samples corresponding to ~1 × 10^4^ cells. Value for DNA sample infected with each heat-inactivated control was shown at 0 h post-infection as a background (**B**). The efficiency of 1^st^ strand-transfer (**C**), (+) strand synthesis (**D**) and 2^nd^ strand-transfer (**E**) at each time point after infection were estimated based on the qPCR analysis as described in Materials and Methods. Means ± SE in duplicate assays are shown. Statistical significance was assessed by student *t* analysis (***: p < 0.001). HIV-1 gene expression from proviral DNA was determined by measuring luciferase activity (luc activity) in the cell lysate at 20 hr post-infection of each virus (**F**). Relative luc activity normalized to the level of U3/gag at 20 h post-infection was calculated. Values were shown as a relative ratio to the relative luc activity of WT as 1.0. These experiments were performed three times and representative experiments with means ± SE in duplicate assays are shown. Statistical significance was assessed by student *t* analysis (n.s.: not significant).

Next, we examined impact of changing the G1-form frequency on the early events of HIV-1 infection. For this infection experiment, virus preparation containing ~80 ng of p24 was inoculated into 293T cells. After 2 h, culture medium was replaced with fresh medium to remove unbound viruses. Then, infected cells were cultured and harvested at 2, 4, 8, 12 and 20 h post-infection. Total DNA isolated from each cell fraction was subjected to qPCR analysis to determine the levels of HIV-1 cDNA intermediates during reverse transcription (Fig. 3A and Material and Method). The levels of R/u5 (-sscDNA), U3/u5 (1^st^ strand-transfer) and U3/pbs (+sscDNA) produced by both viruses were quickly increased and reached peak at 4–8 h post-infection, suggesting -sscDNA synthesis, 1^st^ strand-transfer and +sscDNA synthesis occurred quite efficiently after infection (Fig. 3B). Meanwhile, the level of U3/gag (2^nd^ strand-transfer) showed slower kinetics reaching peak level at 8–12 hr post-infection (Fig. 3B: U3/gag), suggesting the 2^nd^ strand-transfer was rate-limiting step during reverse transcription. Based on this qPCR analysis, we estimated efficiencies of strand-transfer events by each virus (Material and Method). Of note, virus produced from the 5′-G1 clone (5′-G1) showed significantly higher efficiency of the 1^st^ strand-transfer (p < 0.001) compared with the control virus (WT) from parental HIV-1 clone (Fig. 3C). Importantly, the 1^st^ strand-transfer of WT virus was estimated to 60–70 % over time. These results indicated that ~30% of -sscDNA generated by WT virus failed during 1^st^ strand-transfer. The efficiency of 1^st^ strand-transfer in each virus was well correlated with the frequency of the G1-form of HIV-1 RNA within virus particle (Figs 1 and 2). Meanwhile, the later events of +sscDNA synthesis and 2^nd^ strand-transfer did not differ between the both viruses (Fig. 3D,E). These results demonstrated an advantage of HIV-1 RNA with G1-form during 1^st^ strand-transfer. We also examined viral gene expression at 20 h post-infection. Viral gene expression was determined by measuring luciferase activity relative to the late cDNA products (U3/gag) level, which represented completion proviral DNA after successful 2^nd^ strand-transfer. No significant difference was observed between two viruses (Fig. 3F). This result indicated that neither nuclear transport nor integration of viral DNA was affected by the 5′-G on viral genomic RNA. Taken together, we demonstrated the biological advantage of the G1-form of HIV-1 RNA during reverse transcription, specifically at the 1^st^ strand-transfer step.

### Reconstitution of HIV-1 reverse transcription *in vitro*

**Figure 4:**
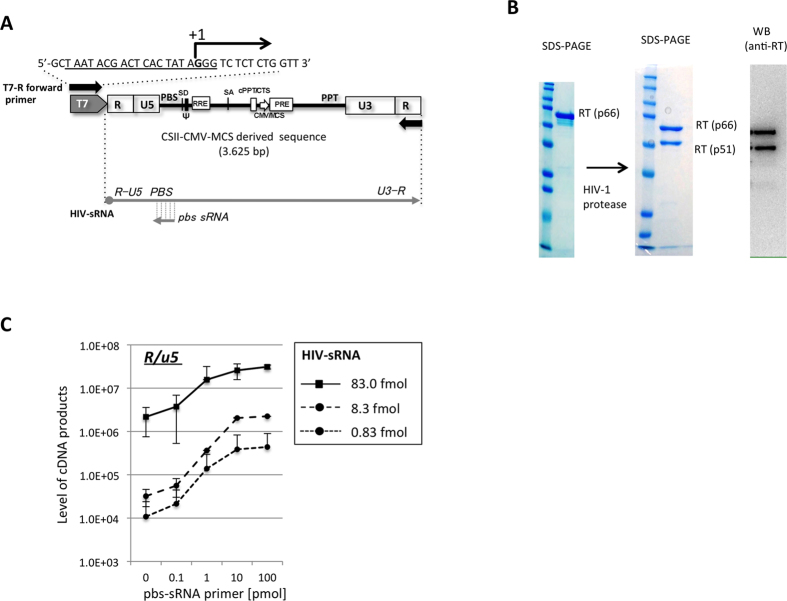
Reconstitution of HIV-1 reverse transcription *in vitro*. (**A**) Genomic information of the synthetic HIV-1 RNA (HIV-sRNA, 3625-nt) was depicted on the top of figure. The nucleotide sequences of T7-R forward primer were shown with the transcriptional initiation site (+1). The promotor sequences for T7 polymerase were underlined. The HIV-sRNA initiates at 5′ end of R region in the 5′ LTR and terminates at 3′ end of R region in the 3′ LTR. Locations of the primer binding site (PBS) and polypurine tract (PPT) in the transcript was indicated. Other cis-elements derived from the lentiviral vector were also indicated; splice donor site (SD), packaging signal (Ψ), rev responsive element (RRE), splicing acceptor site (SA), central polypurine tract/central termination sequence (cPPT/CTS), cytomegalovirus promoter/multiple cloning site (CMV/MCS) and woodchuck hepatitis virus posttranscriptional regulatory element (PRE). (**B**) Recombinant HIV-1 RT (rRT) in heterodimeric form (p66/p51) was generated by proteolysis of the rRT in homodimer form (p66/p66) with HIV-1 protease. Final preparation of the p66/p51 was subjected to SDS-PAGE followed by staining with coomassie brilliant blue and western blot analysis with anti-HIV-1 RT antibody. (**C**) Primer-dose dependency of the cDNA synthesis was examined with three different amounts of HIV-sRNA (0.83, 8.3 or 83 fmol) and 1.7 pmol of rRT. The level of R/u5 in two μl of diluted reaction sample after 90 min incubation at 42 °C was determined by qPCR. The experiment was performed in duplicate for each reaction and the mean value with error bar of ± standard deviation (S.D.) was plotted.

To address molecular basis for the biological advantage of the G1-form of HIV-1 RNA during reverse transcription, we established *in vitro* reconstitution assay for HIV-1 reverse transcription. For this assay, we generated synthetic HIV-1 RNAs (HIV-sRNA) by T7 RNA polymerase using HIV-1 derived lentiviral vector, pCSII-CMV-MCS^28^ as a template (Fig. 4A). The T7 *in vitro* transcription generated HIV-sRNA (~3.6 kb) contained all of the minimum cis-elements required for HIV-1 reverse transcription (R, PBS and PPT) and integration spanning from the 5′-R region to 3′-R region. Because of promotor sequences commonly used for the T7 RNA polymerase (Fig. 4A), the HIV-sRNA had three G at 5′-end (G3-form). The heterodimeric form (p66/p51) of recombinant HIV-1 RT (rRT) was prepared by treatment of the purified rRT in form of p66 with HIV-1 protease (Fig. 4B). The HIV-sRNA was reacted with 1.7 pmol of rRT (p66/p51) and synthetic RNA (18nt) complementary to the HIV-1 PBS sequence (pbs-sRNA) as a primer. Under this condition, the R/u5 product that represents synthesis of -sscDNA was generated in dose-dependent manners of pbs-sRNA ranging from 0.1 to 100 pmol and HIV-sRNA ranging from 0.83 to 83 fmol per 20 μl of reaction (Fig. 4C).

### Quantitative and qualitative analysis of *in vitro* synthesized cDNA intermediates

**Figure 5:**
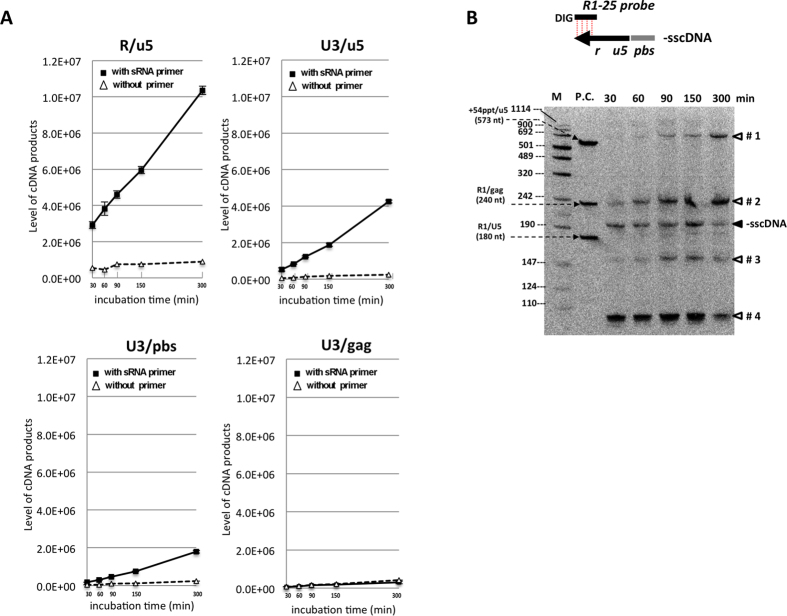
Time course analysis of cDNAs and strand transfer *in vitro*. (**A**) *In vitro* RT assay was performed with 68 fmol of HIV-sRNA, 100 pmol of pbs-sRNA primer and 1.7 pmol of rRT in 20 μl of reaction volume at 42 °C for 30, 60, 90 or 300 min (■). The reaction without pbs-sRNA was carried out in parallel (△). Levels of the cDNA intermediates (R/u5, U3/u5, U3/pbs and U3/gag) were determined by qPCR. Values are shown as copy numbers of the cDNA intermediates in two μl of diluted reaction sample. The experiment was performed in duplicate for each reaction and the mean value with error bar of ±1S.D. of each sample was shown. (**B**) Aliquot of each reaction for the time-course analysis was subjected to Southern blot analysis under denatured condition. DIG-labeled HIV-1 primer R1-25 (DIG-R1-25 probe) was used to detect -sscDNA and its extended products. The -sscDNA and pbs-sRNA were shown by black arrow and grey bar. The minus-strands corresponding to the R, U5 and PBS regions were indicated with small characters of r, u5 and pbs, respectively (top). The bands corresponding to -sscDNA (200 nt) and abortive cDNA products (#1–#4) were indicated. DIG-labeled DNA size marker (DNA Molecular Weight Marker VIII, Roche) was used as a size marker (M). The PCR products that span 54 nt upstream of the PPT to 5′-end of the U5 (+54ppt/u5, 573 nt), the R to 5′-end of gag (R1/gag, 240 nt) and the R to 5′-end of U5 (R1/u5, 180 nt) regions of pCSII-CMV-MCS vector were used as positive controls (P.C.) to test specificity for a DIG-probe.

To evaluate whether the synthesis of -sscDNA and subsequent strand-transfer events occurred sequentially in our reaction, levels of the cDNA intermediates were determined by qPCR using HIV-1 specific primer pairs (Fig. 3A and Material and Method). Levels of R/u5 (-sscDNA) were increased linearly with a fast kinetic during the incubation time up to 300 min (Fig. 5A). Levels of U3/u5 (1^st^ strand-transfer) were increased sequential to the level of R/u5. The levels of U3/pbs (+sscDNA) were increased over time but with a slower kinetic compared to the level of U3/u5. The synthesis of +sscDNA depends on the extension of minus-strand synthesis of -sscDNA at least to the PPT region. The slower kinetics of the U3/pbs reflected partly a poor or incorrect extension of -sscDNA after 1^st^ strand-transfer.

To verify the synthesis of -sscDNA and its derivative products, aliquot of each reaction used for the time course analysis were subjected to Southern blot analysis. The DIG-labeled R1-25 probe was used to detect the -sscDNA (Fig. 5B). The band with expected size of the -sscDNA (200 nt) was clearly detected at 30 min. Then, the level of -sscDNA was fluctuated during the next 120 min and dramatically reduced at 300 min of incubation. Dramatic reduction of -sscDNA at 300 min may reflect the continuous strand-transfer of -sscDNA, some of which was represented by several extra bands accumulated over time (Fig. 5B, denoted by arrow-heads with #1–4). Importantly, these extra bands except #4 were completely absent when reaction was performed without pbs-sRNA primer or when reaction was performed with rRT lacking RNase H activity (Supplementary Fig. S1A). In addition, these extra bands of #1, #2 and #3 reacted with the AA55 probe that was designed to hybridize with plus-strand of the U5 region of HIV-1, while the band corresponding to -sscDNA did not (Supplementary Fig. S1B). These results indicated that extra bands of #1, #2 and #3 were generated by improper inter- or intra-annealing of -sscDNA after RNase H digestion. Within -sscDNA of HIV-1, there are GGG triplets at four different positions (Supplementary Fig. S2). The extra band of #2 (~250 nt), which was generated most abundantly as early as 30 min, might be generated through interaction of the 3′-CCC of -sscDNA with one of the GGG triplet followed by subsequent elongation to 5′-end of the pbs-sRNA. To support this notion, we successfully isolated one of the abortive cDNA that might be generated through the interaction of the 3′-end of -sscDNA with the GGG triplet at position IV within U5 region (Supplementary Fig. S2). Taken together, our *in vitro* assay revealed that more than 70% of -sscDNAs generated from the G3-form of HIV-1 RNA failed the 1^st^ strand-transfer.

### Impact of G number at 5′-end of RNA on reverse transcription *in vitro*

**Figure 6:**
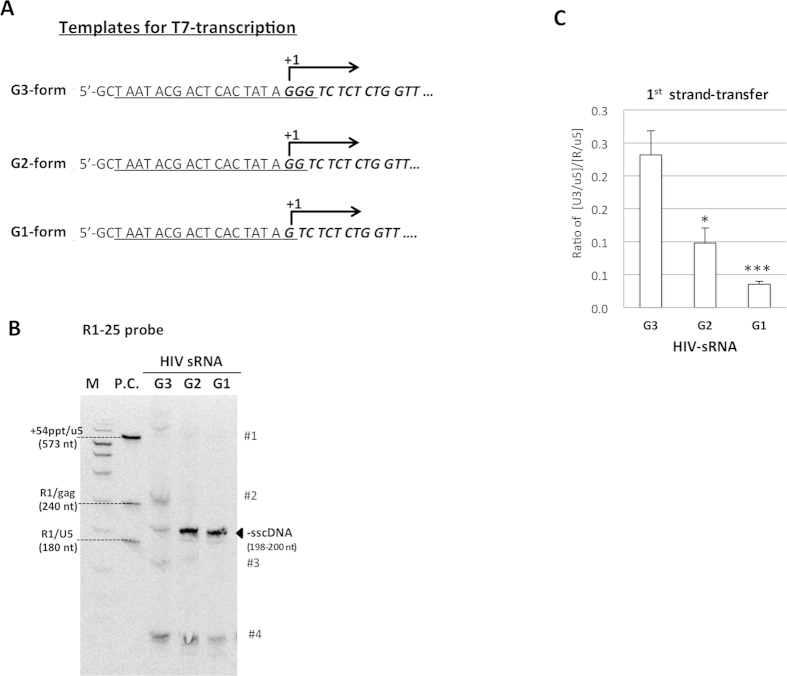
Evaluation of the 5′-G on strand-transfer by *in vitro* reverse transcription assays. (**A**) HIV-sRNAs containing one (G1-form), two (G2-form) or three Gs (G3-form) at their 5′-ends were synthesized *in vitro* using HIV-1 DNA templates prepared with the modified T7-R forward primers. The nucleotide sequences corresponding to the T7-promotor region were underlined. Italics represented 5′-terminal nucleotide sequences of each HIV-sRNA. +1: the transcriptional initiation site. (**B**) *In vitro* reverses transcription was performed with HIV-sRNA with G1-, G2- or G3-form for 300 min, followed by Southern blot analysis using the DIG-R1-25 probe. The band corresponding to -sscDNA (198-200 nt) and abortive cDNA products (#1-#4) were denoted. DNA size marker (M) and positive controls (P.C.) were run together as described in Figure 5. (**C**) The efficiency of 1^st^ strand-transfer in the reaction with HIV-sRNA with G1-, G2- or G3-form was estimated by qPCR analysis as described in Materials and Methods. The experiment was performed in duplicate for each reaction and the mean value with error bar of ±1S.D. of each sample was shown. This experiment performed at least three times and representative result was shown. Statistical significance was assessed by student *t* analysis (*p < 0.05, ***p < 0.001).

Next, examined impact of G number at 5′-end of HIV-sRNA on the reverse transcription events by using *in vitro* reconstitution assay. We generated HIV-sRNA with G1- or G2-form by using template prepared with modified T7-R forward primers (Fig. 6A). Numbers of G at 5′-end of each HIV-sRNA was confirmed by 5′-RACE analysis. Interestingly, levels of the abortive products derived from -sscDNAs (#1–#3) were significantly reduced and the bands corresponding to the -sscDNAs (199 or 198 nt) were stably detected when HIV-sRNA with G2-form or G1-form was used for the *in vitro* reaction (Fig. 6B). On the other hand, efficiency of 1st strand-transfer was significantly reduced with the G1- or G2-form of HIV-sRNA compared with that with the G3-form (Fig. 6C). The q-PCR analysis estimated that the 1st strand-transfer efficiency was less than 5% in the reaction with the G1-form. These results strongly suggested that the number of G at 5′-end of HIV-1 RNA was a critical determinant to regulate generation of abortive products after -sscDNA synthesis.

### Impact of NC on the strand-transfer

**Figure 7:**
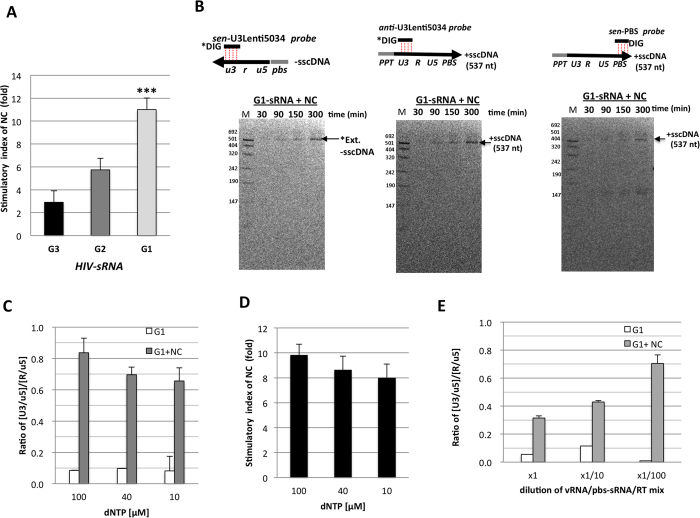
Stimulatory effects of HIV-1 NC on 1^st^ strand-transfer. (**A**) The G1-form of HIV-sRNA (68 fmol) and pbs-sRNA (100 pmol) were pre-incubated with sNC (10 pmol) for 5 min at 37 °C. Pre-incubation without sNC was carried out in parallel as a control. Reaction was initiated by adding the reaction mixture containing 1.7 pmol of rRT (p66/51). After incubation for 300 min at 42 °C, each reaction was subjected to the qPCR analysis. Efficiency of 1^st^ strand-transfer was estimated as described in Fig. 3. The ratio of the 1^st^ strand-transfer efficiency in the presence of sNC to that without sNC was shown as stimulatory index. Experiment was performed in duplicate for each reaction and the mean value with error bar of ±1S.D. of each sample was shown. This experiment performed at least three times and representative result was shown. Statistical significant was assessed by student *t* analysis (***p < 0.001). (**B**) Time course analysis of the cDNAs generated from the G1-form of HIV-sRNA with sNC was addressed by Southern blot analysis using the DIG-R1-25, DIG-anti-U3Lenti5034 or DIG-senPBS probe. The bands corresponding to –sscDNA (200 nt), +sscDNA (537 nt), the abortive products (#1–#4) and extent products of -sscDNA after successful 1^st^ strand-transfer (*EP) were denoted. DNA size marker (M) and positive controls (P.C.) were run together as described in Fig. 5. *In vitro* reverses transcription was performed with G1-form of HIV-sRNA in reaction buffer containing 10, 40 or 100 μM of dNTPs. The levels of cDNA intermediates in the reaction at 300 min were determined by qPCR. The efficiency of 1^st^ strand-transfer (**C**) and stimulatory effect of sNC (**D**) was shown. *In vitro* reverses transcription was performed with serial 10-fold dilution of G1-form HIV-sRNA (68 pmol), pbs-sRNA (100 pmol) and rRT (1.7 pmol) complex in the presence or absence of sNC. The efficiency of 1^st^ strand-transfer of each reaction was presented (**E**). Experiments performed in triplicates. Significant of stimulatory effect of sNC was examined by student *t* analysis (***p < 0.001). nd: not determined because the amount of the U3/u5 was under detection level.

Previous studies have revealed critical roles of HIV-1 nucleocapsid protein (NC) during the strand-transfer events through its nucleic acid chaperone activities (for review see ref. 17). Next, the impact of NC on the strand-transfer efficiency was evaluated under our *in vitro* assay condition. Synthetic HIV-1 NCp7 (sNC) that has been shown to possess the RNA chaperone activity^29^ was used in the following experiments. In the pilot experiments, we noticed that sNC significantly stimulated 1^st^ strand-transfer in Zn^2+^ independent manner but decreased the level of -sscDNA when amount of NC was exceeded 15 pmoles per 20 μl of reaction (Supplementary Fig. S3). Based on this pilot experiment, we used 10 pmoles of sNC (0.5 μM in final concentration) in the following experiments. Of note, we found that stimulatory effect of sNC on 1^st^ strand-transfer was the highest (>10-fold) when reaction was performed with the G1-form of HIV-sRNA (Fig. 7A). Time course analysis of cDNA intermediates confirmed that accumulation of bona fide extension products of the -sscDNA (Fig. 7B). Judging from the size, the -sscDNA might be extended to PPT region after successful 1^st^ strand-transfer. Concomitantly, accumulation of other bands corresponding to +sscDNA (537 nt) was detected by anti-U3lenti5043 probe, which was designed to hybridize with plus-strand U3 region. The band corresponding to +sscDNA (537 nt) also reacted with sen-PBS probe that was designed to hybridize with plus-strand PBS region. These results demonstrated that the 1^st^ strand-transfer and subsequent extension of the -sscDNA and +sscDNA synthesis were successfully reconstituted with G1-form of HIV-sRNA in the presence of NC. We reproduced significant stimulatory effect of NC on 1^st^ strand-transfer under reaction with 10 μM dNTPs corresponding to the concentration within activated T cells (Fig. 7C,D) and with 10- or 100-fold dilution of the G1-form of HIV-sRNA, pbs-sRNA and rRT concentrations (Fig. 7E). These results suggested that impact of sNC on the G1-form of RNA *in vitro* might have a physiologic relevance. Taken together, *in vitro* reconstitution assay revealed that number of G at 5′-end of HIV-1 RNA contributed to successful 1^st^ strand-transfer by reducing abortive cDNA generation of -sscDNA.

## Disucussion

Here, we demonstrated an unprecedented role of 5′-G number on HIV-1 genomic RNA for successful 1^st^ strand-transfer during reverse transcription. HIV-1 transcription initiation site defines the 5′-G number of HIV-1 genomic RNA. Menees *et al*., have demonstrated that G1-form of HIV-1 RNAs was the major form in both of virus particle and producer cells^20^. Our results were consistent with this previous report in that HIV-1 genomic RNAs packaged in virus particles were preferentially in the G1-form (Fig. 1B,C). However, we detected other forms of viral RNAs with additional 5′-Gs in cells persistently infected with HIV-1 or transfected with HIV-1 clone DNA. These results suggested that HIV-1 transcripts were efficiently transcribed at 1 or 2 nt upstream of the currently proposed initiation site. This alternative initiation site was consistent with the other previous study using S1 nuclease mapping method^22^. We also detected unexpected transcripts with deletions or additional Gs (G4-form) at their 5′-ends. We could not deny the possibility that these unexpected transcripts might be generated during cDNA synthesis and subsequent amplification procedures of the 5′ RACE analysis. In this study, therefore, we removed these uncertain products from the data. To address possible linkage of the 5′-G number with splicing event, we generated cDNA pools by using several HIV-1 specific primers that hybridized to upstream or downstream region of the major splice donor (SD) site to distinguish un-spliced form of transcript from other spliced forms. The low frequency of the G1-form in the cellular mRNA fraction was constantly reproduced in each cDNA pool, suggesting the 5′-G number might be independent from the splicing events of HIV-1 mRNA. Nonetheless, it would be interesting to examine the frequency of the 5′-G in other cell types to identify host factors that affect the HIV-1 transcription initiation site.

In this study, we found that the substantial amount of abortive cDNAs was generated during 1^st^ strand-transfer when the G3-form of HIV-1 sRNA was used for the *in vitro* reaction. Analysis of de novo synthesized cDNAs *in vitro* showed that an intra- or inter- annealing of the -sscDNA might be a major cause to produce abortive cDNA products (Supplementary Fig. S2). Meanwhile, it has been reported that HIV-1 RTs can perform template switches even with a very short (2-nt) complementarity between the donor strand and the DNA or RNA template acceptor strands^30^. This RT-induced strand 'clamping' activity might also contribute generation of abortive cDNA products. Several studies have suggested that the trans-activation response (TAR) hairpin in the R region^31^, dimerization initiation sequences (DIS) hairpin in 5′-leader region^32^ and/or 5′-gag region^33^ might be involved to facilitate the 1^st^ strand-transfer. It would be, therefore, interesting to examine the impact of 5′-G number on HIV-1 RNA structure. Preliminary analysis of CentroidFold structure prediction^34^ suggested that the 5′-G number might affect the 5′-untranslated leader (5′-UTR) region that contains sequences required for dimer-formation and packaging of HIV-1 genomic RNA^35^ (Supplementary Fig. S4). Although the exact structure of each form of HIV-1 RNA remains to be determined, this prediction might partly explain the advantage of the G1-form HIV-1 RNA for packaging into virus particle (Fig. 1) and successful 1^st^-strand transfer during reverse transcription (Fig. 3 and 7).

Regardless of the advantage for packaging and revers transcription, the G1-form of HIV-1 mRNA was expressed at low frequency from HIV-1 provirus (Fig. 1). We would speculate that the highly conserved GGG tract in HIV-1 proviral DNA (http://www.hiv.lanl.gov, HIV Sequence Compendium 2014) might have evolved to adapt HIV-1 promoter to host transcription machinery of RNA polymerase II complex. Indeed, HIV-1 gene expression from the modified 5′-G1 HIV-1 DNA clone was significantly reduced (Fig. 2C,D). We also noticed that the 5′-G1 DNA mutation affected p24 expression more severely compared to the level of luciferase marker gene expression. Therefore, viral mRNAs with additional 5′-G (G2- and G3- forms) might also contribute to achieve an optimal balance of HIV-1 gene expression, in which different splicing-forms of viral mRNAs were used for protein synthesis in a complex manner. As a result, HIV-1 promoter might produce the G3-form of mRNA dominantly, which might, however, not be an optimal form as a template for reverse transcription. Subsequently, selective packaging system of the G1-form might have evolved for accurate and efficient reverser transcription of viral mRNA to spread HIV-1 to other cells with high efficiency.

Under our *in vitro* assay condition, NC significantly stimulated 1^st^ strand-transfer. The stimulatory effect of NC was Zn^2+^ independent (Supplementary Fig. S3). Therefore, major functions of NC reproduce in our assay might be exerted mainly through its basic nature^36^,^37^,^38^. On the other hand, we noticed that NC decreased significantly the level of -sscDNA when amount of NC was exceeded at a certain level (15 pmol per 20 ul of reaction). This threshold of the NC under this condition corresponded to be ~100 molecules per viral RNA. This value corresponds to about 1/5 of the level NC required for binding to viral RNA every seven nucleotides^39^. This may support the notion that NC exerts the helix destabilizing effect on viral RNA not uniformly. Recent study using the selective 2′-hydroxyl acylation analyzed by primer extension (SHAPE) technology revealed that helix destabilizing effect by NC was detected in the first 185 nt located between 5′-end and the PBS region of viral RNA^40^. Taken together, a balance of the two properties of NC, helix destabilization and hybridization promoting activities is critical for efficient reverse transcription events.

Lysyl-tRNA synthetase (LysRS) has been identified as a host factor to facilitate the selective packaging of tRNA^lys3^ 41. Recently, tRNA anti-codon-like element (TLE) in HIV-1 genomic RNA has been identified^42^. TLE also contributes for an annealing of tRNA^lys3^ to primer binding site (PBS) located adjacent to the 5′ R-U5 region of viral RNA^13^,^14^,^43^,^44^. It would be interesting to test purified tRNA ^lys3^ in our *in vitro* assay. Our preliminary data showed that reaction primed with tRNA^Lys3^ also contribute to reduce abortive cDNA generation (data not shown). In this study, we demonstrated that 5′-G of HIV-1 RNA had a critical role for a successful 1^st^ strand-transfer in the context of viral replication cycle. Our results indicated that in addition to NC and tRNA, the 5′-G might function as a cis-regulatory element to avoid abortive cDNA generation.

Primal object of this study was reconstitution of HIV-1 reverse transcription *in vitro*. The 1^st^ strand-transfer and subsequent synthesis of plus-strand cDNA were reproduced sequentially in a single *in vitro* reaction. In contrast to these early events of reverse transcription, the later events including the 2^nd^ strand-transfer were hardly reproduced under current *in vitro* condition. In the context of HIV-1 infection, time course analysis of de novo synthesized cDNAs clearly showed that 2^nd^ strand-transfer was rate-limiting steps during reverse transcription within cell. Therefore, host cell factors^45^,^46^,^47^ might be involved for efficient second strand-transfer and subsequent events to complete reverse transcription. Recently, abortive reverse transcription has been reported to trigger a massive T cell depletion by HIV-1^18^,^19^. However, an intrinsic mechanism for abortive reverse transcription remains to be determined, emphasizing importance of delineation of the mechanism. Our finding also suggested that the 5′-G number might be one of the factors to induce abortive reverse transcription. The *in vitro* reconstitution assay using the G1-form of HIV-sRNA would serve as a useful assay to evaluate additional cis- and trans-acting factors to reproduce entire reverse transcription.

## Material and Methods

### Preparation of HIV-1 mRNAs

Total cellular mRNAs expressed in human T cell line persistently infected with HIV-1IIIB strain, Molt-4/IIIB cells^23^ were isolated by using Isogen-LS reagent (Isogen, Nippon Gene, Toyama, Japan) followed by oligo-(dT) column purification (Oligotex™-dT30, Takara & Clontech Laboratories Inc. Japan). For preparation of HIV-1 genomic RNAs in virus particles, culture supernatant of Molt-4/IIIB cells was harvested and filtered through 0.45-μ-pore-size filters. Then, virus particles in the culture supernatant were precipitated by using PEG-it system according to the manufacture’s protocol (PEG-itTM virus precipitation solution, System Biosciences, CA, USA). Total mRNA from the virus particles was similarly isolated. To remove the cap moiety of mRNA, each isolated mRNA fraction was treated with Tobacco Acid Pyrophosphatase (TAP, Nippon Gene, Toyama, Japan) in the reaction buffer containing 200 units of TAP, 50 mM sodium acetate, 5 mM EDTA and 10 mM 2-mercaptoethanol for 37 °C for 60 min.

### 5′-rapid amplification of cDNA ends (5′-RACE) analysis

cDNAs of each RNA fraction were generated by using HIV-1 specific M661 primer^42^ (Fig. 3) according to the protocol provided in SMARTer^®^ RACE cDNA Amplification Kit (Takara & Clontech, Japan). Then, cDNAs were amplified with universal primer mix (Takara & Clontech, Japan) and HIV-1 specific BB301 primer to HIV-1 PBS region, followed by cloning into pGEM-T vector to make a cDNA library from each sample. DNA sequences corresponding to the 5′-terminal region of HIV-1 RNA was determined by using HIV-1 specific AA55 primer (Fig. 3).

### Construction of HIV-1 mutant clones

The AatII-SpeI fragment of the pNL43luc∆env vector^24^ containing the 5′LTR region was cloned into pGEM5Zf (+) vector (Promega, USA) for mutagenesis. Two Gs were deleted from the GGG tract to generate proviral DNA clone that possessed only one G at the 5′-U3/R boundary. After confirmation of the mutation by DNA sequence analysis, the AatII-SpeI fragment was inserted back into the pNL43luc∆env vector^24^ to generate 5′-G1 HIV-1 DNA clone.

### Preparation of HIV-1 pseudo-type viruses

293T cells were transfected with pNL43luc∆env vector and VSV-G-expression vector, pHCMVG^25^ using linear polyethylenimine (Polyethylenimine Max., Polysciences, Inc. Warrington, PA). After 24 h, the culture medium were replaced with 5 ml of Duldecco’s modified Eagle’s medium (DMEM) supplemented with 10% fetal bovine serum, 100 μU/ml penicillin and 100 μg/ml streptomycin and incubated for 24 h. The culture supernatant was harvested and filtered through 0.45-μm-pore-size filter and used as virus preparation. The virus preparation was treated with DNase I (100 units/ml; Worthington, Lakewood, NJ) in the presence of 10 mM MgCl_2_ at 37 °C for 40 min to avoid plasmid DNA contamination. An aliquot of the virus preparation was incubated at 65 °C for 30 min and used as a heat-inactivated control. The level of HIV-1 p24 antigen in each virus preparation was determined using an enzyme immunoassay (HIV-1 p24 ELISA kit, Ryukyu Immunology Corp. Okinawa, Japan). For luciferase analysis, transfected cells were harvested at 48hr post-transfection. The cells (~1 × 10^7^ cells) were washed twice with phosphate-buffered saline (PBS) and lysed with 1ml of luciferase lysis buffer (Promega), and 1 μl of each cell lysate was subjected to the luciferase assay.

### Virus infection

For virus infection, an aliquot (corresponding to ~100 ng of p24) of the DNase-treated virus preparation was inoculated into 293T cells (~1 × 10^6^ cells). Total cells were harvested at each time point (2, 4, 8, 12, and 24 h) after infection. After washing the cells with phosphate-buffered saline, nucleic acids were extracted as described previously^48^. Briefly, cells were disrupted in urea lysis buffer [4.7 M urea, 1.3% sodium dodecyl sulfate (SDS), 0.23 M NaCl, 0.67 mM EDTA, pH 8.0] and subjected to phenol-chloroform extraction and ethanol precipitation. The resulting DNA pellet was resolved in 100 μl of TE buffer (10 mM Tris, pH 8.0, 1 mM EDTA, pH 8.0). An aliquot of each sample (~5 × 10^3^ cells equivalent) was subjected to qPCR using primer pairs specific for the R/U5 region of HIV-1 (M667/AA55), the U3/U5 region (U3NL9496/AA55), the U3/PBS region (U3NL9496/BB301) or the U3/gag region (U3NL9496/M661). For luciferase analysis, infected cells were harvested at 20 h post-infection. The cells (~1 × 10^6^ cells) were washed twice with PBS and lysed with 200 μl of luciferase lysis buffer (Promega) and 10 μl of each cell lysate was subjected to the luciferase assay.

### qPCR analysis

The DNA samples diluted with TE were subjected to qPCR analysis using the real-time Light Cycler detection system (Light Cycler Instrument; Roche, Mannheim, Germany). Two μl of each diluted samples were amplified with HIV-1 primer pairs. The primers of AA55, BB301 and M661 have been used for qPCR to estimate the level of reverse transcription intermediates during HIV-1 infection as reported in previous studies^24^,^48^,^49^. The primer of R1-25, U3NL9496 and U3Lenti5034 were newly designed in this study. R1-25 (5′-gtctctctggttagaccagatctga-3′) and AA55 primer pair (R1-25/AA55) used to estimate levels of minus-strand strong stop cDNA (-sscDNA).

U3NL9496 (5′-gctgcatataagcagctgctttttgcct -3′) and was designed to hybridize with U3 region of pNL43luc∆env vector. U3Lenti5034 (5′-gctgcatccggactgtactg-3′) was designed to hybridize with U3 region in 3′LTR of pCSII-CMV-MCS vector. The primer pair of U3NL9496/AA55 or U3Lenti5034/AA55 was used to estimate levels of cDNA intermediates after the first strand-transfer of -sscDNA. The primer pair of U3 U3NL9496/BB301 or U3Lenti5034/BB301 was used to monitor levels of plus-strand strong stop cDNA (+sscDNA). The primer pair of U3NL9496/M661 or U3Lenti5034/M661 was used to estimate levels of cDNAs after the second strand-transfer of +sscDNA. For the standard DNA to estimate copy number, pGEM-T vector (Promega, USA) containing the U3 and gag regions of pCSII-CMV-MCS was generated. A serial dilution of the vector corresponding to 10^2^–10^8^ copies was subjected to the qPCR analysis in parallel. The efficiency of 1^st^ strand-transfer of -sscDNA was estimated by calculating the relative amount of U3/u5 to that of R/u5 (% of [U3/u5]/[R/u5]). Similarly, efficiency of +sscDNA synthesis and 2^nd^ strand-transfer were estimated by relative amount of U3/pbs to that of U3/u5 (% of [U3/pbs]/[U3/u5]) and by relative amount of U3/gag to that of U3/pbs (% of [U3/gag]/[U3/pbs]), respectively.

### Preparation of synthetic HIV-1 RNA

The DNA region (~3.5 kbp) of HIV-1 lentivirus vector (pCSII-CMV-MCS, RIKEN BioResource Center, Tsukuba, Japan^28^) spanning from the R regions in 5′ LTR to the R region in 3′ LTR was amplified by PCR using the T7-R forward primer (5′-GCT AAT ACG ACT CAC TAT AGG GTC TCT CTG GTT AGA CCA GAT CTG A-3′) and R-end reverse primer (5′-TTG AGC ACT CAA GGC AAG CTT TAT TGA GGC-3′). The T7-R forward primer contains the minimum T7 RNA polymerase promoter sequence as shown with underline. To generate HIV-1 RNA with one (G1) or two guanines (G2) at 5′-termini, modified T7-R forward primer of 5′-GCT AAT ACG ACT CAC TAT AGG TC TCT CTG GTT AGA CCA GAT CTG A-3′ or 5′-GCT AAT ACG ACT CAC TAT AG TC TCT CTG GTT AGA CCA GAT CTG A-3′ was used, respectively. The obtained PCR fragment contains minimum cis-elements required for reverse transcription and integration of HIV-1. The fragment was purified through agarose gel electrophoresis and used for subsequent *in vitro* transcription reaction as a template. HIV-1 RNA was prepared by the T7 *in vitro* transcription system (RiboMAX^TM^ Large Scale RNA Production systems, Promega, CA, USA) according to the manufacture′s protocol. After the *in vitro* transcription, template DNA was carefully removed by DNase I digestion (TURBO DNase, Ambion, USA) and each transcript was purified using ISOGEN LS kit (Nippon Gene, Japan) followed by the gel filtration using NAP-5 column (GE Healthcare, USA). Peak fractions were gathered and precipitated with 70% ethanol. The pellet of the transcript was dissolved in DEPC-treated deionized distilled water. Size of each transcript was verified by electrophoresis in denatured condition. The initiation site of the transcript was confirmed by 5′-rapid amplification of cDNA ends (5′-RACE analysis) using SMARTer^®^ RACE cDNA Amplification Kit (Takara & Clontech, Japan).

### RNA primers

The synthetic RNA (18nt) complementary to the HIV-1 PBS sequence (pbs-sRNA) 5′-gucccuguucgggcgcca-3′ was synthesized and purified by HPLC (SIGMA Aldich Japan Genosis).

### Preparation of recombinant RTs

The entire RT (p66) region of HIV-1 (pNL4-3) was cloned into pET-47b(+) vector (Novagen, USA). His-tagged form of HIV-1 RT p66 (His-RTp66) was expressed in the *E. coli* BL21 derivative strain of Rosetta (DE3) (Invitrogen) by culture with 1 mM IPTG for 4 hr at 30 °C. His-RTp66 was purified through the Ni-NTA column (QIAGEN, USA). Then, His-tag was removed by treatment of His-RTp66 with Turbo3C protease (Nacalai-Tesque, Japan) and subjected to ion exchange chromatography with SP-column (GE Healthcare). Fractions eluted by 1xSP buffer [20 mM HEPES-NaOH (pH7.0), 0.1 mM EDTA, 0.01% TritonX-100, 10% glycerol and 1 mM DTT] containing 250–500 mM NaCl were pooled. The purified recombinant HIV-1 RT (rRT-p66) was stored in 1xSP buffer containing 500 mM NaCl and 40% glycerol at −80 °C. To obtain the heterodimeric (p66/p51) form of HIV-1 RT, purified rRT-p66 was treated with HIV-1 protease (PROSPEC, Israel) in PR-buffer [20 mM Tris-HCl (pH7.0) and 1 M NaCl] for 1-2 hr at 37 °C^50^. After the protease treatment, heterodimeric form of rRT (p66/p51) was concentrated through a concentrator column with the filtration cut-off of 100 kDa (VIVAspin 100K, GE Healthcare, USA). The heterodimer form of rRT was confirmed by SDS-PAGE analysis and western blot analysis using anti-HIV-1 RT antibody (Abcam, Japan). Conventional assay measuring incorporation of [^3^H]-dTTP into the oligo-dT/poly(A) substrate estimated that the purified HIV-1 RT (p66/p51) possessed 80–100 units/μg. Retroviral RT (M-MLV RT) lacking RNase H activity was purchased from TOYOBO Life Science (ReveTra Ace, TOYOBO Life Science, Japan).

### Synthetic HIV-1 NC

A lyophilized sample of synthetic nucleocapsid protein NCp7 (LAV strain 72-aa sequence) was purchased from Peptide Institute Inc. (Osaka, Japan). The chaperone activity of the synthetic NC (sNC) on HIV-1 RNA dimerization was confirmed in the previous study^29^. Pre-treatment of NC with ZnCl_2_ was performed by incubation of 0.4 mM sNC in the 0.8 mM ZnCl_2_ for 10 min at room temperature. The sNC stock (0.4 mM) with or without 0.8 mM ZnCl_2_ was serially diluted with deionized distilled water and used for the *in vitro* reverse transcription assay.

### *In vitro* reverse transcription assay

Synthetic HIV-1 RNAs (HIV-sRNA) was denatured at 75 °C for 5 min and immediately cooled on ice to avoid inter- and intra-molecular annealing of HIV-1 sRNA. Then, heat-denatured HIV-1 RNA was mixed with pbs-sRNA primer in a final volume of 20 μl of 1xRT buffer [50 mM Tris-HCl (pH8.3) 75 mM KCl, 3 mM MgCl_2_, 10 mM DTT, 0.1 mM dNTPs] on ice. Reaction was initiated by adding 1 μl of rRT diluted by 1xRT buffer containing 0.07–8.7 pmol of rRTs. To examine effect of synthetic NC, rRT (1.7 pmol), HIV-1 sRNA (68 pmol) and pbs-sRNA (100 pmol) were pre-incubated with sNC for 5 min at 37 °C. Reaction was performed at 42 °C for 30–300 min and terminated by heating at 95 °C for 5 min.

### Southern blot analysis

Each reaction sample was mixed with an equal volume of sample buffer (95% Formamide and 0.05% BPB). After incubation at 95 °C for 5 min, the sample was run on 8% denaturing polyacrylamide gel containing 8M Urea. DNAs in the gel were electrically transferred into nylon membrane in 0.5× TBE buffer (40 mM Tris-HCl, pH 8.3, 45 mM boric acid and 1 mM EDTA). Viral cDNAs on the membrane was detected by hybridization with a digoxigenin (DIG)-labeled HIV-1 specific probe according to the protocol supplied by the DIG Nucleic Acid Detection Kit (Roche). Images of the hybridization were obtained by using a chemiluminescence imaging system (Chemilum ImageQuant Las 400mini, GE Healthcare, Japan). DIG-labeled DNA size marker (DNA Molecular Weight Marker VIII, DIG-labeled, Roche) and PCR products that span 54 nt upstream of the PPT to 5′-end of the U5 (+54ppt/u5, 573 nt), the R to 5′-end of gag (R/gag, 240 nt) and the R to 5′-end of U5 (R/u5, 180 nt) regions of pCSII-CMV-MCS vector were used as a size marker and positive controls for each DIG-probe, respectively.

## Additional Information

**How to cite this article**: Masuda, T. *et al.* Fate of HIV-1 cDNA intermediates during reverse transcription is dictated by transcription initiation site of virus genomic RNA. *Sci. Rep.* **5**, 17680; doi: 10.1038/srep17680 (2015).

## Supplementary Material

Supplementary Figures
