# Supplementary material for: Fate of HIV-1 cDNA intermediates during reverse transcription is dictated by transcription initiation site of virus genomic RNA
Source: Sci Rep. 2015 Dec 3;5:17680. doi: 10.1038/srep17680 (PMC4668388; doi:10.1038/srep17680)
Supplement: Supplementary Figures [file srep17680-s1.pdf]

SUPPLEMENTARY INFORMATION

Fate of HIV-1 cDNA intermediates during reverse transcription is dictated by transcription initiation site of virus genomic RNA

Takao Masuda[1,*], Yoko Sato[1], Yu-Lun Huang[1], Satoshi Koi[1], Tatsuro Takahata[1], Atsuhiko Hasegawa[1], Gota Kawai[2] and Mari Kannagi[1]

[1]Department of Immunotherapeutics, Graduate School of Medical and Dental Sciences, Tokyo Medical and Dental University, Yushima, Bunkyo-ku, Tokyo 113-8519, Japan

[2] Department of Life and Environmental Sciences, Faculty of Engineering, Chiba Institute of Technology, 2-17-1 Tsudanuma, Narashino-shi, Chiba 275-0016, Japan

* To whom correspondence should be addressed. Tel: +81-3-5803-5799; Fax: +81-3-5803-0235; Email: tmasu.impt@tmd.ac.jp

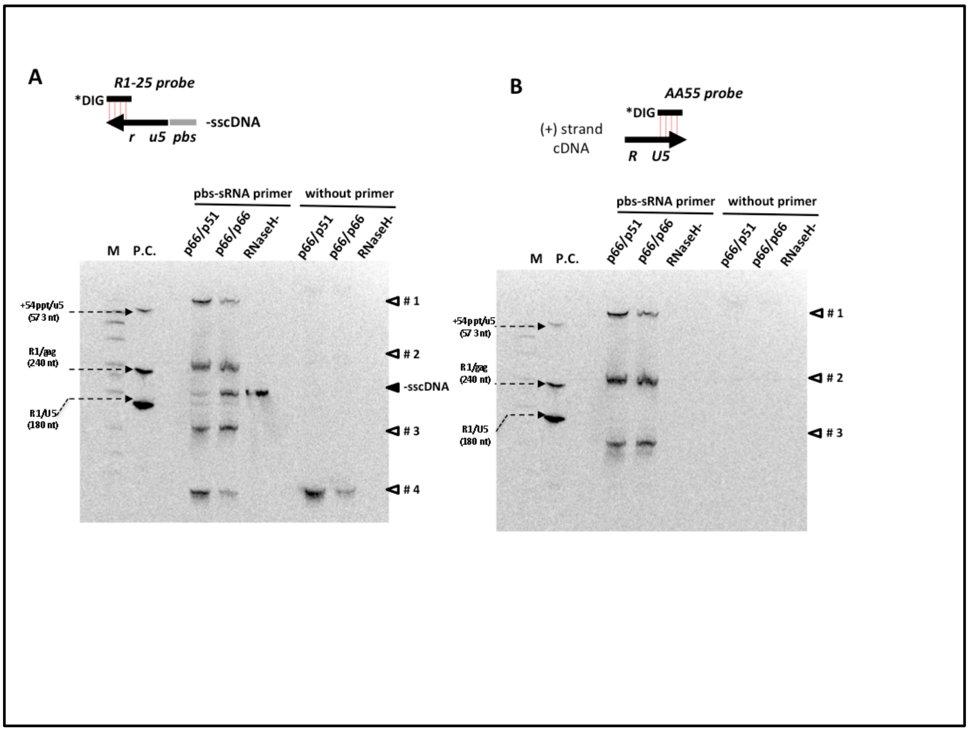

Supplementary Figure. S1: Southern blot analysis of cDNA intermediates. (A) In vitro reverse transcription was performed in the same condition as described for Figure 5 by using HIV-1 rRT (p61/p51), homodimer (p66/p66), or M-MLV RT lacking RNase H activity. Reactions without pbs-sRNA primer (without primer) were carried out in parallel. After 300 min incubation, generation of -sscDNA and the abortive products was examined by Southern blot analysis using DIG-R1-25 probe. (B) The same membrane was re-probed with DIG-AA55 probe to detect the minus-strand of U5 sequences. The bands corresponding to -sscDNA (200 nt) and abortive products were denoted by (#1-#4). DIG-labeled DNA size marker (DNA Molecular Weight Marker VIII, DIG-labeled, Roche) and PCR products that span 54 nt upstream of the PPT to 5'-end of the U5 (+54ppt/u5, 573bp), the R to 5'-end of gag (R/gag, 240 bp) and the R to 5'-end of U5 (R/u5, 180bp) regions of pCSII-CMV-MCS vector were used as a size marker and positive controls to test specificity for each DIG-probe, respectively.

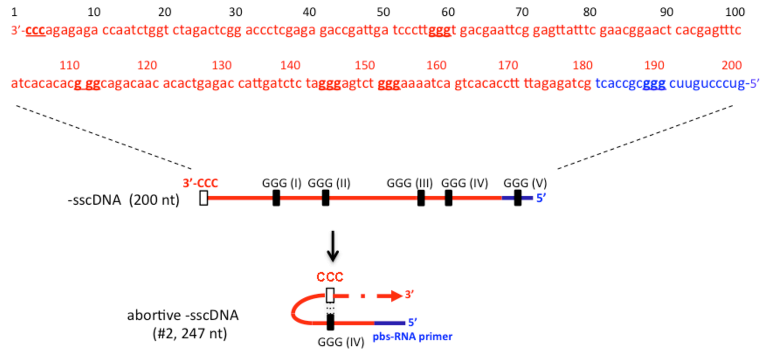

Supplementary Figure S2. Speculative structure of abortive products of HIV-1 -sscDNA. Nucleotide sequences of HIV-1 -sscDNA (200nt) were shown. The -sscDNA with three cytosines (CCC) at 3′-end was generated from the G3-form of HIV-1 RNA. There are four (I-IV) GGG triplets (bold letters) within the -sscDNA, location of which were shown with black bars. Schematic picture of the abortive product #2, which might be generated through interaction of the 3′-end CCC with the GGG triplet at position IV, was drawn. Expected size of the abortive product that extended to the end of the pbs-primer by fold-back of the 3′-CCC with the GGG triplet (IV) was shown.

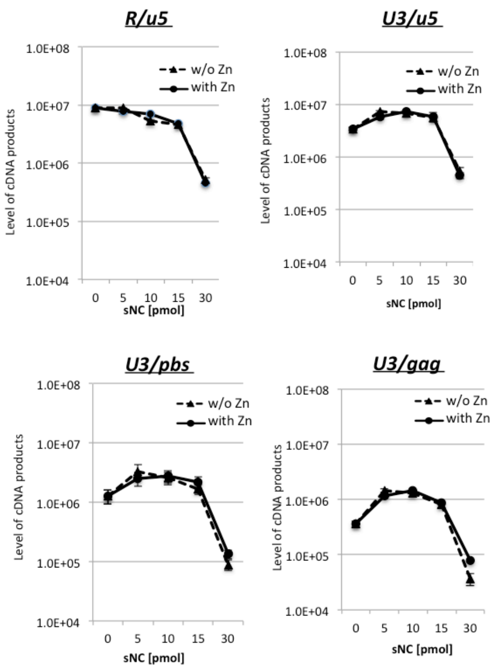

Supplementary Figure S3. Effect of HIV-1 NC on cDNA intermediates in vitro. HIV-sRNA (68 pmol) and pbs-sRNA (100 pmol) were pre-incubated with serial dilutions of sNC (0, 5, 10, 15 or 30 pmol) by deionized and distilled water for 5 min at 37˚C. The same serial dilutions of sNC that were pre-treatment of with $ZnCl_2$ (with Zn) or without $ZnCl_2$ (w/o Zn) were examined in parallel. Reaction was initiated by adding the reaction mixture containing 1.7 pmol of rRT (p66/51). After incubation for 300 min at 42˚C, each reaction was subjected to qPCR analysis. Values were shown as copy number of R/u5, U3/u5, U3/pbs or U3/gag in 2 µl of each diluted sample. The experiment was performed in duplicate for each reaction and the mean value with error bar of ± S.D. of each sample was shown.

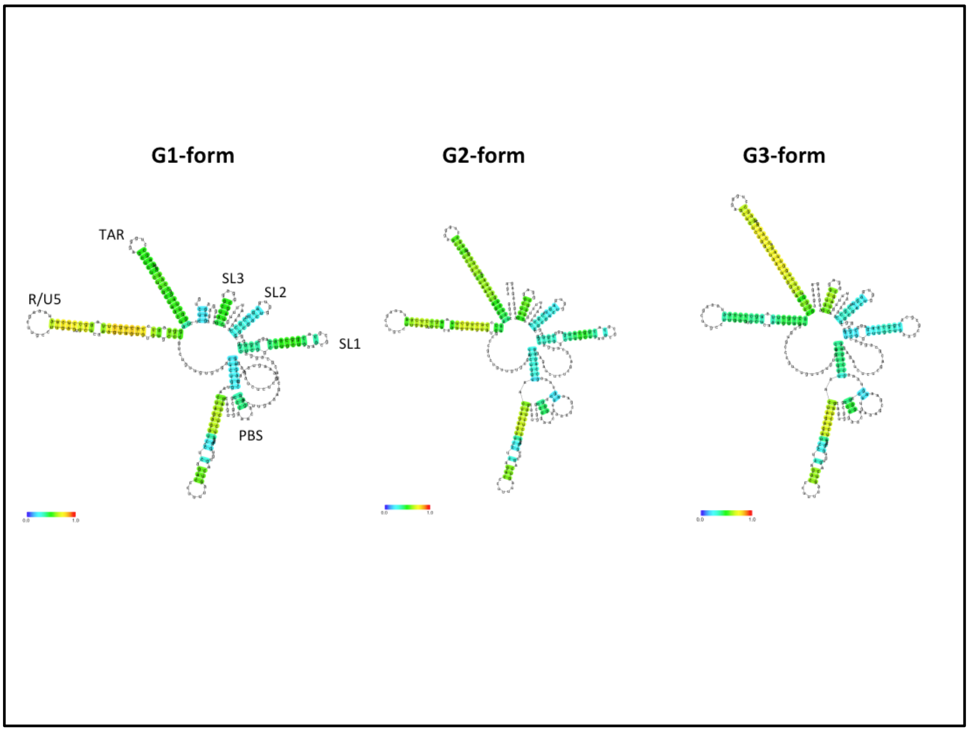

Supplementary Figure S4. RNA structure prediction for HIV-1 5'-untranslated leader region with different number of the 5'-G. Impact of 5'-G number on HIV-1 RNA structure of the 5'-untranslated leader (5'-UTR) region was predicted by using CentroidFold (http://www.ncrna.org/centroidfold). Predicted structures of HIV-1 5'-UTR with one (G1-form), two (G2-form) and three 5'-Gs (G3-form) were shown. The trans-activation response (TAR), R/U5, primer binding site (PBS) and three stem-loop (SL1-SL3) in the 5'-UTR were indicated. Each predicted base-pair is colored with the heat color gradation from blue to red corresponding the base-pairing probability from 0 to 1.
